# Heterogeneous responses in Google Trends measures of well-being to the COVID-19 dynamic quarantines in Chile

**DOI:** 10.1038/s41598-022-18514-z

**Published:** 2022-08-25

**Authors:** Fernando Díaz, Pablo A. Henríquez, Diego Winkelried

**Affiliations:** 1 Departamento de Ingeniería Comercial, Universidad Técnica Federico Santa María, Santiago, Chile; 2 School of Business and Economics, Universidad Diego Portales, Santiago, Chile; 3 Center for Empirical Research in Businesses (CIEN), Universidad Diego Portales, Santiago, Chile; 4 School of Economics and Finance, Universidad del Pacífico, Lima, Peru

**Keywords:** Health policy, Epidemiology

## Abstract

We study how the Chilean population’s well-being responded to the strategy implemented by their health authorities, known as *Dynamic Quarantine*, to contain the spread of coronavirus in which municipalities periodically entered and exited lockdowns. This unique scheme, together with the population’s socioeconomic heterogeneity, facilitates the estimation of changes in this well-being as differentiated by socioeconomic status. Using Google Trends to compute measures of well-being, we find strong evidence that socioeconomic status induces heterogeneity in these changes; thus, neglecting this heterogeneity may lead to misleading prescriptions for the public policy that addresses the psychological effects of lockdowns.

## Introduction

In response to the outbreak of the COVID-19 pandemic, governments around the globe declared health emergencies and imposed various restrictions to reduce the mobility of the population, such as through curfews and lockdowns, to curb the spread of the virus. In addition, most countries implemented the largest fiscal and monetary stimulus packages in their histories to tackle the great economic damage of the health measures.

This context of great health, economic, and social uncertainties has shifted the attention of authorities, academics, and the general public to the well-being of the population and the possible consequences of the long periods of lockdown and social distancing on their mental health. Canet-Juric et al.^1^ claim that the lockdowns represented a serious threat to mental health during COVID-19, while Mucci et al.^2^ argue that social isolation has produced feelings such as uncertainty, fear, and despair that affect the mental health of the population: “in the near future, mental health professionals will be called to face a ‘parallel pandemic’ of acute stress disorders, post-traumatic stress disorder (PTSD), emotional disturbance, sleep disorders, depressive syndromes and eventually suicides” (p. 63). Similarly, Casagrande et al.^3^ find low sleep quality, anxiety, and distress to be associated with pandemic-related PTSD. Shah et al.^4^ find that the uncertainty surrounding the pandemic triggered mental health problems, such as anxiety, stress, and depression, and that these feelings deepened with the length of the lockdown.

According to Brodeur et al.^5^, beyond the direct economic costs of the virus containment strategies, there are other considerable costs in terms of social unrest, school disruption, and well-being that need to be accounted for. In an effort to provide an economic rationale for the decision to lift or to maintain lockdowns, Layard et al.^6^ do a cost-benefit analysis of this decision in terms of a well-being metric based on the so-called “WELLBY approach”^7^. They conclude that relieving lockdowns can improve mental health that alleviates the suicide rate, domestic violence, addiction, and loneliness.

In a related strand of recent literature, but not directly assessing the pandemic, Clark et al.^8^ analyze the impact of terrorism on the well-being of the population. They use a measure of well-being that accounts for positive and negative emotions that are associated with everyday activities. Terrorism can affect the daily lives of individuals by increasing feelings of uncertainty, fear, and risk aversion, and these authors find the effects of terrorism on daily activities to be as harmful as a two percentage point increase in annual unemployment. On the other hand, Leigh-Hunt et al.^9^ offer systematic reviews that provide consistent evidence that links social isolation and loneliness to worse mental health outcomes.

In this paper, we analyze the effects of lockdowns on the well-being of the Chilean population. Like most countries, the Chilean government responded stringently to the outbreak of the pandemic. However, unlike the lockdowns imposed elsewhere in which the entire population or specific cities were confined, the Chilean health authorities followed a strategy known as a *Dynamic Quarantine*. This strategy entailed introducing or continuing lockdowns every week in some municipalities and lifting those in others based on their assessment of the epidemiological situation^10,11^. The selective and asynchronous lockdown of the population under such a scheme, together with a high degree of socioeconomic heterogeneity among municipalities, provide a unique opportunity to measure the differential effects that lockdowns could have on the well-being of populations belonging to different socioeconomic statuses (SES). Our empirical approach benefits from the fact that in the Chilean case, a single health authority decides the lockdown status of all municipalities in the country; if these decisions were made by different authorities, as happens in cross-country comparisons, the identification of the effects of the lockdowns on the well-being of the population could be hindered by confounding factors.

**Figure 1:**
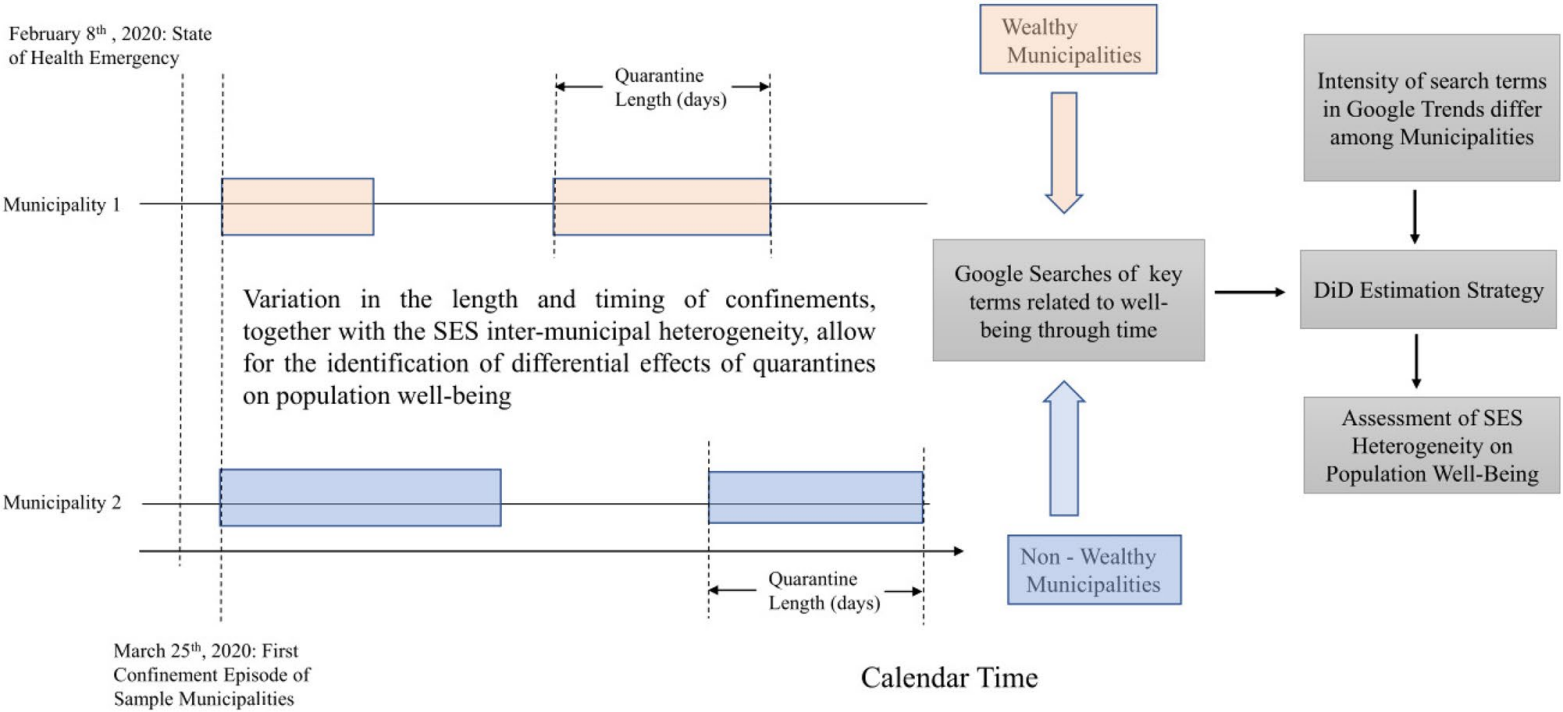
Estimation strategy. The diagram represents the length and timing of lockdowns under the Chilean *Dynamic Quarantine* scheme. Wealthy and non-wealthy municipalities are represented by different colors. Calendar time is on the horizontal axis and the length of the boxes for each municipality represents the duration of the lockdowns. The beginning or end of a lockdown need not be similar among municipalities. DiD refers to “difference-in-differences”.

Most of the growing body of research on the evolution of mental well-being throughout the pandemic have based its empirical methods on surveys and online questionnaires. However, as Brodeur et al.^5^ point out, to have an adequate assessment of how the pandemic and related government responses have affected the population, having data prior to the pandemic is crucial. Thus, in their key contribution to the literature, they circumvent the problem of the lack of a benchmark measure of well-being by using historical internet keyword searches from Google Trends (GT). GT provide aggregate measures of search activity in a location (e.g. a state or country) that studies have widely used to examine these economic and social phenomena: namely, well-being^5^, unemployment^12^, employment^13–15^, stock trading behavior^16^, migration^17^, tourism flows^18^, and disease outbreaks^19^ like COVID-19^20^. Recently, Wang et al.^21^ provide evidence that suggests that GT may be a valid novel epidemiological tool to map depression prevalence. Following Brodeur et al.^5^, we base our analysis on the responses of GT-based measures of well-being to the containment measures and the general state of the pandemic. Then, we use a difference-in-differences (DiD) estimation strategy to assess the hypothesis that SES matters in the sense that the responses of GT-based well-being measures are statistically different between wealthy and non-wealthy populations. In Fig. 1 we provide a diagrammatic representation of our empirical framework.

We contribute to the literature in several ways. First, we add to the relatively few papers that have investigated the causal effect between lockdowns and well-being^5,22,23^. To the best of our knowledge, this is the first study that estimates the differential effects of lockdowns on different SES segments of the population using non-survey data. Instead, we use GT to measure the interest in topics or search terms related to well-being. Second, we provide evidence that the population’s heterogeneity in SES influences the changes in its well-being during lockdowns. In other words, we find that in a number of cases, the responses from inhabitants of wealthy municipalities, as reflected in their searches in GT, differ from those of non-wealthy municipalities. This result has important implications for both policymakers and researchers. In the first place, it eloquently indicates that it is crucial to incorporate such heterogeneity in the design of policies aimed to support the population in dealing with PTSD as a result of the COVID-19 pandemic^9^. In the second place, any attempt to measure a population’s well-being might yield biased results if SES heterogeneity is not controlled for.

## Methods

### Measuring well-being

Our measure of well-being is based on GT searches. As described in Scott and Varian^13^, GT is a service that produces time series data on search volume intensity (SVI) to measure the popularity of a particular keyword in a specific period and location. The SVI is measured on a scale that ranges from 0 to 100, where the value of 100 indicates the peak of popularity and 0 indicates complete disinterest. GT may qualify analyzed phrases as either a search term or a topic. Search terms are literally typed words, while topics may be proposed by GT when the tool recognizes phrases related to popular queries. While collecting data, values below 1, denoted as “$< 1$”, were replaced by 1. We specify the region as CL (Chile).

**Table 1:** Search terms used for searches on Google Trends.

Topic	Keywords (Searches in Spanish)	Keywords in English
*Boredom*	Aburrimiento, Tedio, Fastidio, Aburrido, Aburrida, Qué lata, Qué fome	Monotony, Dullness, Doldrums, Tedium, Tiresomeness, Wearisomeness
*Loneliness*	Soledad, Aislamiento, Solo, Sola, Abandono, Incomunicación, Incomunicado, Separación, Quiebre, Ausencia, Encierro, Encerrado, Encerrada	Solitude, Isolation, Lonesomeness, Separation, Solitariness, Loneliness, Alienation, Friendlessness, Lonely Feeling, Feeling Alone
*Irritability*	Irritabilidad, Irritable, Mal genio, Intolerante, Impaciente, Intolerancia	Irascibility, Irritation
*Panic*	Pánico, Miedo, Susto, Asustado, Asustada	Alarm, Anxiety, Awe, Consternation, Desperation, Dismay, Dread, Fear, Scare
*Sleep*	Dormir, Dormir bien, Insomnio, Desvelo, Devalado, Desvelada	Snooze, Rest, Doze, Repose, Siesta, Nap, Catnap, Hibernation
*Stress*	Estrés, Estresado, Estresada, Tensión, Intranquilidad, Nerviosismo, Nervioso, Nerviosa, Intranquilo, Intranquilidad, Ansiedad, Ansioso, Ansiosa	Anxiety, Pressure, Nervousness, Tension, Nervous Tension, Concern, Uneasiness, Agony, Burden, Distress
*Worry*	Preoucpación, Inquietud, Inquieto, Inquieta, Problema, Aproblemado, Aproblemada, Cuidado, Molestia, Molesto, Molesta, Nerviosismo, Nervioso, Nerviosa, Angustia, Angustiado, Angustiada, Depresión, Deprimida, Deprimido, Culpa, Culposo	Be Worried, Fret, Be Bothered, Be Anxious, Brood
*Frustration*	Frustración, Frustrado, Frustrada, Impotencia, Apestado	Annoyance, Anger, Resentment, Disappointment, Discomfiture, Dismay, Chagrin, Dissatisfaction, Detdown
*Self-care*	Autocuidado, Sobrepeso, Obesidad, Obeso, Obesa, Gordo, Gorda, Gordura	Care, Health Care, Maintenance Mental Health, Care Personal, Care Self-aid, Self-help

We Google search for nine well-being and mental health related topics for the months from March 2020 to July 2020 and for the same months in 2019. These topics, presented in Table 1, were taken from Brodeur et al.^5^ who in turn choose topics that are as close as possible to the different items in the *General Health Questionnaire*^24,25^. A growing number of studies in psychology have explored the psychological effect of lockdowns and personal quarantines during the COVID-19 outbreak. Their list of words on psychological effects resembles our list of topics^8,9,26–29^. Since the actual searches were made in Spanish, we provide the 87 Spanish keywords that are associated with these topics in the second column of Table 1. As a reference, we present these keywords in English in the third column of the same table.

The SVI of the proposed keywords are aggregated by taking the average across all individual keywords within a topic for each day *t* to obtain an average search volume intensity, $\mathrm {ASVI}_{t}$. The higher the value of the ASVI, the greater the population’s attention to that topic on a specific day. Then, we follow the work of Da et al.^30^ by using the abnormal search volume activity (ASVA) as our proxy for the well-being of the population. The ASVA is defined as:

1 $$\begin{aligned} \mathrm {ASVA}_{t}= \ln \left( \frac{\mathrm {ASVI}_{t}}{\mathrm {ASVI}_{t}^*} \right) \,. \end{aligned}$$

where $\ln (.)$ denotes the natural logarithm, and $\mathrm {ASVI}_{t}^*$ is computed as the monthly average of the corresponding ASVI during the corresponding month in 2019. Thus, the ASVA is the log-percent deviation in the current search volume intensity from a reference value in a normal year. Table 2 presents the descriptive statistics for the ASVA that we computed between January and August, 2020. We also include the descriptive statistics for the COVID-19 reproductive number $R_0$ that is used later in our empirical specifications to control for the general conditions of the pandemic.

### Lockdowns and socioeconomic status

The Chilean *Dynamic Quarantine* has very peculiar characteristics which have already attracted the interest of researchers^10,31,32^. There are two distinct stages in the Chilean strategy. In the early stage, which corresponds to the period between March 24th and July 20th, 2020, the government imposed complete lockdowns in different municipalities according to their pandemic situation. In the second stage, the so-called *step by step plan*, the government changed its strategy to ease the complete lockdowns imposed up to that point by implementing restrictions based on five stages or incremental steps that ranged from lockdown to advanced opening. We restrict our attention to the first stage in which the health authorities made a total of 25 weekly announcements (available at www.minsal.cl) about which municipalities nationwide would be under lockdown during the following week.

We consider the lockdown situations of municipalities with a population larger than 13,000 people. This sample covers 120 municipalities that represent approximately 83% of the country’s total population (14.5 million people). The descriptive statistics for the number of lockdowns and for the number of days in their duration are presented in Table 3. As a proxy for the SES of the population, we use the poverty index (PI) reported by the Ministry of Social Development (Ministerio de Desarrollo Social y Familia) in the CASEN 2017 household survey (available at http://observatorio.ministeriodesarrollosocial.gob.cl). Following Díaz and Henríquez^31^, we consider wealthy municipalities as those in the top decile of the PI sorting of which there are 12 that comprise 1,858,618 inhabitants, which is roughly 11% of the country’s total population. This figure is close to the 12% that corresponds to the proportion of people who belonged in the top socioeconomic segment in 2018, according to the Association of Market Researchers and Public Opinion of Chile. Our final sample that includes these 12 municipalities and non-wealthy ones totals 117 for which we have complete SES information.

**Table 2:** Descriptive statistics: ASVA and the reproductive number.

	Mean	Median	St. Dev.	Min	Max
Boredom	0.003	0.16	0.73	− 2.60	1.27
Loneliness	0.09	0.11	0.30	− 0.73	0.82
Irritability	− 0.05	0.03	0.53	− 1.53	1.32
Panic	0.28	0.35	0.50	− 1.18	1.24
Sleep	0.14	0.10	0.48	− 1.05	1.47
Stress	0.10	0.13	0.34	− 0.91	0.92
Worry	0.02	0.01	0.28	− 0.84	0.76
Frustration	− 0.20	0.31	1.31	− 2.12	2.02
Self-care	0.12	0.17	0.35	− 1.24	1.05
$R_0$	1.24	1.30	0.26	− 0.91	1.86

**Table 3:** Number of lockdowns and days under Lockdown.

	Whole Sample		Not Wealthy municipalities		Wealthy municipalities	
(A) Number of lockdowns						
0	48		46		2	
1	61		56		5	
2	8		3		5	
Total	117		105		12	

	Whole Sample		Not Wealthy municipalities		Wealthy municipalities	
Days under lockdown	First	Second	First	Second	First	Second
(B) Days under lockdown						
Mean	34.08	35.31	34.96	36.12	26.36	35
Max	118	91	118	91	69	69

### Effects on well-being

Next, we describe the framework for our empirical analysis to study the effects of lockdowns on the well-being of the population, as measured by the ASVA index, and their heterogeneity across municipalities classified by wealth. Such effects can be consistently estimated in a linear equation with interactions in what is known as a difference-in-differences (DiD) estimation^33–37^. To be more precise, the nature of the dynamic lockdown scheme means different treatment timing for different municipalities. This setup corresponds to a *staggered DiD* model that is similar to the event study method that uses *event time* (number of days since the announcement of a lockdown) rather than calendar time^38,39^. However, in our empirical framework, the dependent variable, ASVA, is already a difference between the volume of searches between day *t* and an historical average; thus, the estimation we entertain is actually a triple difference estimation - DiDiD (time, lockdown status, and wealth)^40,41^.

Municipalities are the smallest administrative unit in Chile and, geographically, they constitute a finer partition than is covered by GT. In other words, for each day *t* in our sample period, we observe the same value of the ASVA for all municipalities. We discuss the conditions under which the effects of interest, and their heterogeneity across municipalities, can be estimated consistently despite this measurement particularity.

#### Effects of interest

Let *i* index municipality, *t* index calendar time, and $\tau$ be a counter of the number of days since the announcement of the last lockdown. Further, the $\tau$ resets to zero when the end of the lockdown if finally announced. Given the dynamic nature of the Chilean lockdown strategy, $\tau$ depends on *t* but to alleviate the notation we leave that dependence implicit.

We define a dummy variable $D_{it}(\tau )$ such that $D_{it}(\tau ) = 1$ if municipality *i* at time *t* has been locked down for $\tau$ periods (i.e., if municipality *i* is locked down at time *t*, a condition that was announced at calendar time $t - \tau$), and $D_{it}(\tau ) = 0$ otherwise. Let $Y_{it}$ be the ASVA for a given topic or search term related to some dimension of well-being in municipality *i* at time *t*. Then, we have:

2 $$\begin{aligned} \Delta (\tau ) = \mathrm {E}(Y_{it} \mid D_{it}{(\tau )}=1) - \mathrm {E}(Y_{it} \mid D_{it}{(\tau )}=0)\,, \end{aligned}$$

that is the average effect of a lockdown on the well-being after $\tau$ periods for the population in any Chilean municipality. Thus, the profile $\{\Delta (1), \Delta (2), ..., \Delta (m)\}$ is of interest to track the evolution of such effects as the lockdown continues over *m* periods.

Let $W_i$ be a municipality-specific dummy variable such that $W_i = 1$ if municipality *i* is wealthy, and $W_i = 0$ if it is not. This dummy variable is not time varying since we do not expect a change in the socioeconomic status of municipalities in the relatively short time span of our sample. Then, for $k=\{0,1\}$, we define:

3 $$\begin{aligned} \Delta _k(\tau ) = \mathrm {E}(Y_{it} \mid D_{it}{(\tau )}=1, W_i = k) - \mathrm {E}(Y_{it} \mid D_{it}{(\tau )}=0, W_i = k) \,, \end{aligned}$$

as the average effect of a lockdown, after $\tau$ periods, on the well-being measure of the inhabitants of a wealthy $(k = 1)$ or a non-wealthy $(k = 0)$ municipality. We define:

4 $$\begin{aligned} \Delta _{10}(\tau )= \Delta _1(\tau ) - \Delta _0(\tau )\,, \end{aligned}$$

as the differentiated lockdown effect on well-being in a wealthy municipality relative to that in a non-wealthy one. While $\Delta (\tau )$ in (2) has an effect on the average municipality, $\Delta _{10}(\tau )$ in (3) indicates if such an effect depends on the socioeconomic status. Thus, the profile $\{\Delta _{10}(1), \Delta _{10}(2), \ldots , \Delta _{10}(m)\}$ is of interest to study whether the lockdown produces this gap over *m* periods.

Let $p = \text {Pr}(W_i=1)$ be the proportion of wealthy municipalities in Chile at the time of the pandemic. Then, the average effect of a lockdown on well-being in the population can be written as:

5 $$\begin{aligned} \Delta (\tau ) = p \,\Delta _1(\tau ) + (1-p)\Delta _0(\tau ) = \Delta _0(\tau ) + p \, \Delta _{10}(\tau ) \,. \end{aligned}$$

which relates the effects on subpopulations with the aggregate. Moreover, even though $\Delta (\tau )$ can be identified from the data under mild conditions, the requirements to identify $\Delta _{10}(\tau )$ are more stringent; thus, the decomposition in (5) is not generally feasible. In fact, $\Delta (\tau )$ can be computed from time variation, that is, a before-after comparison with a lockdown that treats all municipalities equally, or from cross-sectional variation as long as there are municipalities that are never affected by the lockdown. On the other hand, $\Delta _{10}(\tau )$ requires variation in both dimensions simultaneously, which is provided by the unique Chilean quarantine design.

#### Regression setup

Studies have well-documented that a DiD estimation can be implemented in a linear regression model that features the treatment dummy variable and its interaction with time. Likewise, a third dimension such as SES can be introduced through further interactions^41^. Thus, the above quantities of interest can be estimated as coefficients in a linear regression, or their linear combinations, which is quite convenient for the computation of standard errors and subsequent hypothesis testing.

Consider a longitudinal sample of *n* municipalities over *T* periods, and the linear regression model:

6 $$\begin{aligned} Y_{it}=\alpha _{i} + \sum _{\tau =0}^{m} \delta _{\tau }D_{it}{(\tau )} +\sum _{\tau =0}^{m} \beta _\tau W_{i}\cdot D_{it}{(\tau ) } \ + \gamma _i \,W_{i}+ \varepsilon _{it}\,, \quad i = 1, 2, \ldots , n; \quad t = 1, 2, \ldots , T\,, \end{aligned}$$

where $(\beta _\tau ,\delta _\tau )$ for $\tau =1,2,\ldots ,m$ are regression coefficients, $\alpha _i$ is a municipality effect and $u_{it}$ is the error term.

The coefficients $\beta$ and $\delta$ can be estimated consistently with the so-called within-group estimator under the mild condition that $\mathrm {E}(\varepsilon _{it} \mid D_{it}(\tau )=d)=0$ for $d=\{0,1\}$ and $\tau =1,2,\ldots ,m$ or, in other words, that $\varepsilon _{it}$ is mean independent from $D_{it}(\tau )$. This independence happens if there is no feedback from the well-being measures on the decision to declare a lockdown, which we argue to be a reasonable assumption as the related policies predate the public worries on mental health and similar considerations. Further, $\alpha _i$ and $\gamma _i$ are not identified, since they are removed from the equation with the within-group transformation.

It follows from (6) that $\mathrm {E}(Y_{it} \mid D_{it}{(\tau )}=1, W_i = k) = \alpha _{i}+\delta _{\tau }+(\beta _{\tau }+\gamma _i)k$, and $\mathrm {E}(Y_{it} \mid D_{it}{(\tau )}=0, W_i = k ) = \alpha _{i}+\gamma _ik$. Thus:

7 $$\begin{aligned} \Delta _0(\tau ) = \delta _{\tau }\,, \qquad \Delta _1(\tau ) = \delta _{\tau }+\beta _{\tau } \,, \qquad \Delta _{10}(\tau ) =\beta _{\tau }\,. \end{aligned}$$

The coefficient $\delta _\tau$ is the effect of a lockdown on the well-being of the population in a non-wealthy municipality, and $\beta _\tau$ is the added effect for the population in a wealthy municipality, that is, the DiDiD. Thus, from (5) and (7) the effect on the whole population is:

8 $$\begin{aligned} \Delta (\tau ) = \delta _{\tau }+ p \, \beta _{\tau } \,. \end{aligned}$$

#### Measurement error

The ASVA is observed at an aggregate level, not at a municipality level. Yet, it is still possible to estimate the quantities of interest. The idea is that the dependent variable is measured with an error. If the measurement error were classical (i.e., simply a noise), then the error term in the regression will increase its variance with no further consequence. In practice, the measurement error may not be classical; what is required is that it does not correlate with the policy interventions after removing time variation and municipality-specific effects.

To elaborate, consider the factor decomposition:

9 $$\begin{aligned} Y_{it} = Y_t + y_i + \theta _i W_i + u_{it} \,, \end{aligned}$$

where $Y_t$ is a time varying factor, $y_i$ a municipality effect and by construction, $u_{it}$ does not have systematic time or cross-sectional variation: $\mathrm {E}(u_{it} \mid Y_t, y_i)=0$. This equation can be thought of as the measurement equation $Y_{t} = Y_{it} - e_{it}$, where $e_{it}=y_i + \theta _i W_i + u_{it}$ is a non-classical measurement error. But the properties of $u_{it}$, not $e_{it}$, are those relevant for our purposes.

By subtracting $e_{it}$ from (6), we get:

10 $$\begin{aligned} Y_{t}=\tilde{\alpha }_{i} + \sum _{\tau =0}^{m} \delta _{\tau }D_{it}{(\tau )} +\sum _{\tau =0}^{m} \beta _\tau W_{i}\cdot D_{it}{(\tau ) } \ + \tilde{\gamma }_i \,W_{i}+ \tilde{\varepsilon }_{it}\,, \end{aligned}$$

where $\tilde{\alpha }_i = \alpha _i - y_i$, $\tilde{\gamma }_i = \gamma _i - \theta _i$ and $\tilde{\varepsilon }_{it}= {\varepsilon }_{it} - u_{it}$. Equation (10) is the feasible, error-ridden version of (6). The within-group estimation removes all influences from $\tilde{\alpha }_i$ and $W_i$, and the consistent estimation of the coefficients $(\beta _\tau ,\delta _\tau )$ is possible if $\mathrm {E}(u_{it} \mid D_{it}(\tau )=d)=0$ for $d=\{0,1\}$ and $\tau =1,2,\ldots ,m$. An interpretation of this mean independence condition is that if all variation in $Y_{it}$ due to the effects on the lockdown and its continuation is captured by $Y_t$, then the coefficients in (10) are exactly like those in (6). Put differently, the coefficients $(\beta _\tau ,\delta _\tau )$ capture the effects of a lockdown and even its heterogeneity across municipalities as classified by $W_i$ that are important enough to produce time variation in the aggregate well-being.

## Results

Next, we present the results for the within-group (fixed-effect) estimates of the coefficients in equation (10), and some linear combinations, for each of the topics in Table 1. Besides the lockdown dummies and their interactions with the municipality SES dummy, the regression also includes the COVID-19 reproductive number $R_0$ to control for the general conditions of the pandemic, month dummies, day of the week dummies, and a first and second lockdown dummy. Standard errors are clustered at the municipal level.

**Figure 2:**
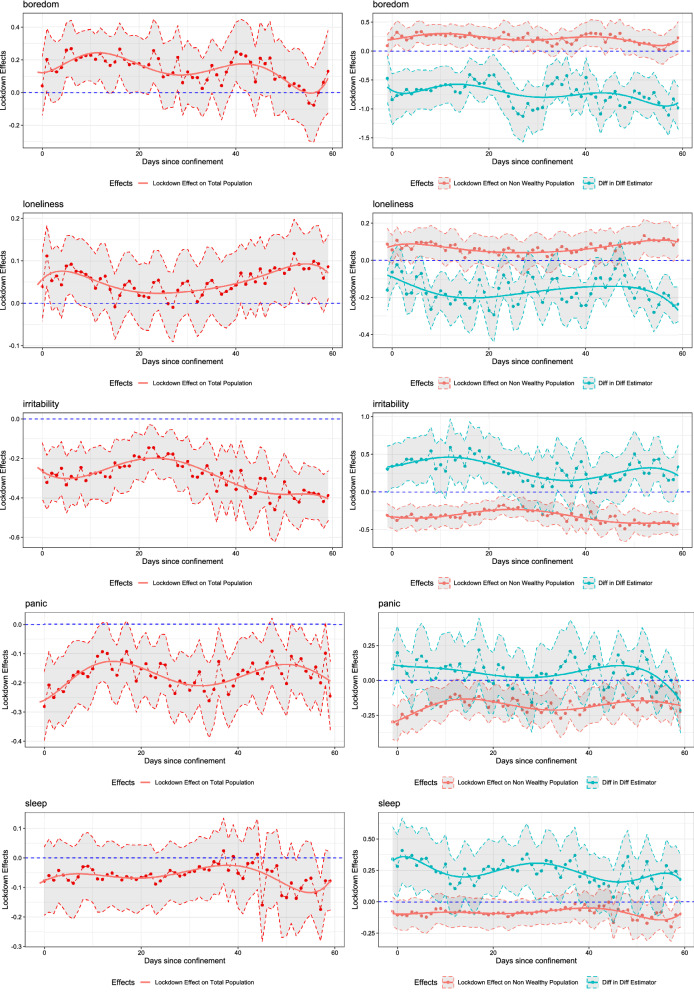

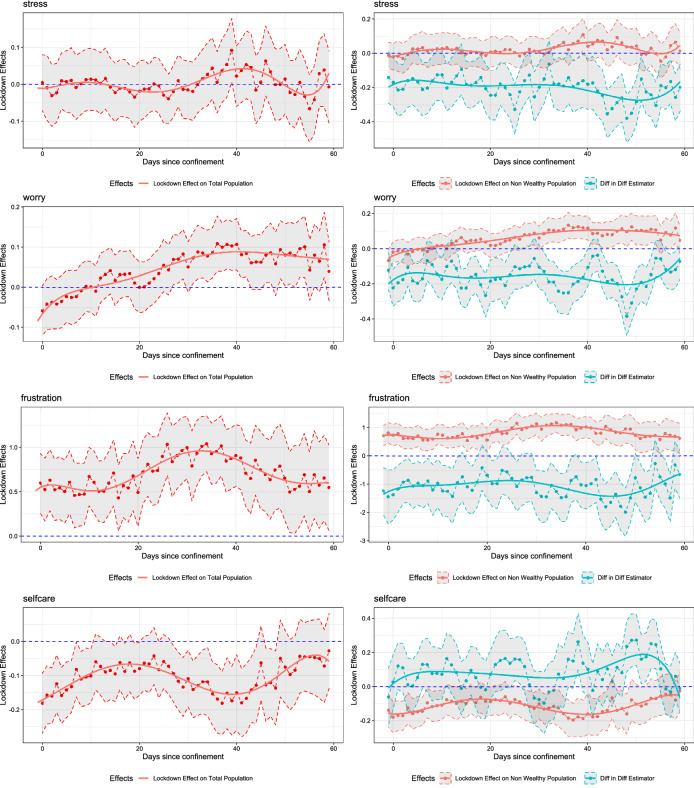
Parameter estimates. Fixed effects estimation of Eqs. (10) and (5). All regressions use $N = 3636$ observations, and control the reproductive number $R_0$ , month and day of the week effects, and the first and second lockdown dummies. To ease visualization, the graphs show the 3-day symmetric moving average of the point estimates of $\Delta (\tau ) = \delta _{\tau }+ p \, \beta _{\tau }$ (left panels), and of $\delta _{\tau }$ and $\beta _{\tau }$ separately (right panels), and their 95% confidence intervals that are based on standard errors clustered at the municipal level. Polynomial trends of the point estimates are also included.

We consider wealthy municipalities as those that belong to the top decile according to the PI sorting, so $p = 0.10$. We also consider $m = 60$ days, so that the effects are up to two months after the announcement of a lockdown. The interest is on the profiles $\{\Delta (\tau )\}_{\tau =0}^{\tau =m}$, $\{\Delta _{0}(\tau )\}_{\tau =0}^{\tau =m}$, and $\{\Delta _{10}(\tau )\}_{\tau =0}^{\tau =m}$ that we are able to estimate without imposing any a priori functional form of $\tau =0,1,\ldots ,m$. Such an eclectic approach provides flexibility in the functional forms but at the cost of abundant outputs which we present concisely in Fig. 2, Tables 4, and 5. In Fig. 2 we present the estimated lockdown effect on the whole population, $\Delta (\tau )$; the lockdown effects on the non-wealthy population, $\delta _\tau$; and the DiDiD estimates, $\beta _\tau$ for $\tau = 0,...,60$ with their corresponding 95% confidence intervals and, to ease visualization, a polynomial time trend. On the other hand, in the tables we report the average estimates across $\tau$, the proportion of periods that the effects are positive or negative, and the corresponding proportion of periods in which the effects are statistically significant (i.e., their confidence intervals do not contain zero) at various confidence levels.

### Effects on the whole population

The panels to the left of Fig. 2 show $\Delta (\tau )$ as a function of $\tau$ for each sentiment. Our first finding is that even though the point estimates do appear to display some time variation, that is, $\Delta (\tau )$ behaves differently for different $\tau$, the differences across $\tau$ are not statistically significant. That is to say, with very few exceptions and for all topics, the confidence interval of some $\Delta (\tau _1)$ overlaps with that of $\Delta (\tau _2)$ for $\tau _1 \ne \tau _2$, which means that we will not be able to reject a null hypothesis of the type $H_0: \Delta (\tau _1) = \Delta (\tau _2)$.

An implication is that in most cases, the effect of an additional day in lockdown on the population’s well-being is relatively constant as $\tau$ increases. It does not increase, nor does it vanish. There are some possible exceptions to this pattern: *Boredom* that indicates some positive and significant short-run effects within the first 20 days; *Worry* whose effect trend upwards and become positive and significant after a month; and *Self-care* whose effect is negative and significant immediately after the announcement and after the first month.

A second finding is that the signs, significances and magnitudes of the effects on the ASVA vary across topics or dimensions of well-being. Table 4 complements Fig. 2 by giving related statistics. The effects on the ASVA of *Sleep* and *Stress* are generally not significant (i.e., their confidence intervals contain zero). For *Sleep*, the average estimated effect is $-0.06$, as nearly 28% of the estimated $\Delta (\tau )$ are positive, with only 6.56% being statistically significant at the 5% confidence level. For the negative ones, only 31% are statistically significant. For *Stress*, the average point estimate of $\Delta (\tau )$ is zero with 52% having positive estimated coefficients of which only 13.1% are statistically significant. The other 48% have negative coefficients of which 18% are statistically different from zero at the 5% level. The results of *Boredom*, *Loneliness*, and *Worry* are similar: the average effects are positive but small (0.15, 0.05, and 0.05, respectively), and the proportion of periods with point estimates that are positive ranges from 70 to 78% out of which between 40 and 51% are statistically significant at the 5% level. For *Self-care* and *Panic*, the average effects on ASVA are negative ($-0.10$ and $-0.18$, respectively). For these topics, the proportion of periods with negative effects are 83 and 86% of which between 50 and 65% are significant at the 5% confidence level. Finally, the effects for *Frustration* and *Irritability* are the most significant and the largest in magnitude, although they are positive for *Frustration* and negative for *Irritability*. The average effects are, respectively, 0.71 and $-0.29$ with more than 95% being positive or negative, respectively, of which between 81 and 84% are significant at a 5% confidence level.

### Socioeconomic heterogeneity

The above effects on the whole population, $\Delta (\tau ) = \delta _{\tau }+ p \beta _{\tau }$, come from combining an effect on the ASVA of non-wealthy municipalities, $\delta _\tau$, with a term proportional to the DiDiD estimator $\beta _\tau$. The parameters $\delta _\tau$ and $\beta _\tau$ can be identified by the unique characteristics of the Chilean dynamic quarantine. The panels on the right of Fig. 2 and Table 5 show the results.

The results on *Boredom*, *Loneliness*, *Stress*, *Worry*, and *Frustration* are similar in that the estimated $\beta _\tau$ are consistently negative, although with various degrees of significance. The negative DiDiD estimates mean that the population from non-wealthy municipalities pays more attention and responds more intensively to these topics during lockdowns than the population from wealthy municipalities. Anecdotally, people in wealthy areas feel less bored, lonely, stressed, worried, and less frustrated as a response to lockdowns than inhabitants in poorer areas.

In the case of *Boredom*, the average $\beta _\tau$ is $-0.74$, and 98% of the coefficients are negative of which 89% are significantly different from zero at a 5% significance level. The average effect on the non-wealthy population, $\delta _\tau$, is 0.22 and the effect is positive in 84% of periods, out of which 57% result significantly different from zero at the 5% level. The $\beta _\tau$ coefficient is large enough (in absolute value) so that these figures are higher and more significant than those corresponding to the whole population (average effect of 0.15 and positive for 79% of the periods of which 51% are significant). A similar configuration is found for *Worry* and *Frustration*, but in the latter case the effects are positive and almost always significant for the whole population. For *Frustration*, the average $\beta _\tau$ is $-1.09$ and this coefficient is significant at the 5% level for 64% of the time for 84% of periods of with negative effects. The average $\delta _\tau$ is 0.82 that is higher than the average $\Delta (\tau )$ of 0.71 but both figures are significant about 80% of the time for more than 95% of the positive effects.

**Table 4:** DiDiD estimation: Lockdown effects on the whole population. Fixed effects estimation of equations (10) and (5): $\Delta (\tau ) = \delta _{\tau }+ p \, \beta _{\tau }$. Standard errors are clustered at the municipal level. All regressions control for the reproductive number $R_0$, month and day of the week effects, and a first and second lockdown dummy. $N = 3636$ observations.

	Boredom	Loneliness	Irritability	Panic	Sleep	Stress	Worry	Frustration	Self-care
Average estimate	0.15	0.05	– 0.29	– 0.18	– 0.06	0.00	0.05	0.71	– 0.10
Positive (%)	78.69	73.77	3.28	13.11	27.87	52.46	70.49	95.08	16.39
**Significant (%)**									
1%	39.34	32.79	0.00	1.64	4.92	4.92	32.79	70.49	1.64
5%	50.82	39.34	0.00	3.28	6.56	13.11	39.34	81.97	1.64
10%	55.74	40.98	0.00	3.28	9.84	19.67	44.26	83.61	3.28
Negative (%)	21.31	26.23	96.72	86.89	72.13	47.54	29.51	4.92	83.61
**Significant (%)**									
1%	6.56	1.64	77.05	57.38	22.95	11.48	6.56	0.00	39.34
5%	11.48	3.28	83.61	65.57	31.15	18.03	9.84	0.00	50.82
10%	14.75	4.92	86.89	72.13	42.62	18.03	9.84	1.64	55.74
$R^2$	0.40	0.30	0.34	0.35	0.38	0.45	0.39	0.30	0.39

**Table 5:** DiDiD estimation: Socioeconomic heterogeneity. Fixed effects estimation of equation (10). Standard errors are clustered at the municipal level. All regressions control for the reproductive number $R_0$, month and day of the week effects, and a first and second lockdown dummy. $N = 3636$ observations.

	Boredom		Loneliness		Irritability		Panic		Sleep		Stress		Worry		Frustration		Self-care	
	$\beta$	$\delta$	$\beta$	$\delta$	$\beta$	$\delta$	$\beta$	$\delta$	$\beta$	$\delta$	$\beta$	$\delta$	$\beta$	$\delta$	$\beta$	$\delta$	$\beta$	$\delta$
Average	– 0.74	0.22	– 0.16	0.07	0.30	– 0.32	0.05	– 0.18	0.25	– 0.09	– 0.20	0.02	– 0.16	0.06	– 1.09	0.82	0.08	– 0.11
Positive (%)	1.6	83.6	19.7	80.3	83.6	0.0	59.0	11.5	83.6	19.7	9.8	59.0	14.8	75.4	16.4	96.7	67.2	11.5
**Significant (%)**																		
1%	0.0	49.2	6.6	36.1	36.1	0.0	19.7	0.0	50.8	3.3	0.0	8.2	1.6	37.7	3.3	73.8	29.5	0.0
5%	0.0	57.4	8.2	42.6	47.54	0.0	26.2	1.6	59.0	4.9	0.0	16.4	1.6	47.5	3.3	78.7	39.3	1.6
10%	0.0	65.6	9.8	47.5	59.0	0.0	31.1	1.6	63.9	6.6	0.0	23.0	1.6	49.2	4.9	85.2	44.3	3.3
Negative (%)	98.4	16.4	80.3	19.7	16.4	100.0	41.0	88.5	16.4	80.3	90.2	41.0	85.2	24.6	83.6	3.3	32.8	88.5
**Significant (%)**																		
1%	85.2	1.6	54.1	0.0	0.0	80.3	11.5	57.4	1.6	24.6	57.4	4.9	47.5	0.0	41.0	0.0	9.8	37.7
5%	88.5	4.9	63.9	0.0	0.0	88.5	11.5	65.6	3.3	41.0	60.7	8.2	57.4	6.6	63.9	0.0	13.1	49.2
10%	88.5	6.6	65.6	1.6	3.3	90.2	14.8	70.5	3.3	57.4	68.9	14.8	63.9	9.8	70.5	0.0	16.4	59.0
$R^2$	0.40		0.30		0.34		0.35		0.38		0.45		0.39		0.30		0.39	

The cases of *Loneliness* and *Stress* are similar in that the $\beta _\tau$ coefficients remain significant, even though they are not large enough to noticeably affect the whole population as compared to the non-wealthy population. For instance, for *Stress* the average $\beta _\tau$ is $-0.20$; this coefficient is negative 90% of the time of which 61% are significant at the 5% confidence level. Correspondingly, the average $\delta _\tau$ is close to the average $\Delta (\tau )$ and their confidence intervals almost always include zero.

On the other hand, the results on *Irritability* and *Sleep* are comparable as the estimated $\beta _\tau$ are mostly significant and positive, that means the population from non-wealthy municipalities respond to lockdowns less intensively in these GT searches than the population from wealthy municipalities. People in wealthy areas tend to feel more irritated and more concerned about sleep. A similar pattern arises for *Self-care* and *Panic* but in general the DiDiD estimates are not significant.

For *Irritability*, the average $\delta _\tau$ coefficients is $-0.32$ in which 88% have significantly negative coefficients at the 5% confidence level. The effects on the whole population are higher. The average $\Delta _\tau$ is $-0.29$ in which the estimated coefficients are negative 97% of the time of which 84% are significant at the 5% level. Thus, the average $\beta _\tau$ is 0.30 which is positive 84% of the time of which 48% are significant. In the case of *Sleep*, the average $\beta _\tau$ is 0.25 which is also positive 84% of the time of which 59% are significant cases.

All in all, these findings disclose important differences between wealthy and non-wealthy populations’ interests in search topics on lockdowns. Furthermore, it provides evidence that the estimations of social well-being during the pandemic might be biased if SES heterogeneity is neglected.

In the estimation of equation (10), we include municipality fixed effects, month time effects, the level of $R_0$, day-of-the-week dummies, and the first and second lockdown dummies. The estimation results for the $R_0$ and for the first and second lockdown dummies are presented in Table 6.

Regarding the $R_0$, we add its level to equation (10) as a control for the general conditions of the pandemic. For most of the topics, we obtain the expected signs for this variable. The higher the $R_0$ is, the higher the levels of *Loneliness*, *Stress*, *Worry*, and *Frustration* in the population are, even though the t-test for *Frustration* barely exceeds a level of one. Interestingly, for *Irritability* and *Sleep*, the estimated $R_0$, coefficient is negative and highly significant. However, during high $R_0$ episodes, the government imposes more stringent measures to control the pandemic, so there are several confounding factors that might be affecting the social well-being.

The estimated coefficient for the second lockdown dummy indicates heterogeneity among the topics. During the second lockdown, people pay more attention and respond more intensively to topics related to *Stress*, *Worry*, and *Frustration*. Interestingly, people are less bored and less irritable during their second lockdown than during their first experience.

**Table 6:** DiDiD estimation: Controls. Fixed effects estimation of the coefficients of control variables in equation (10). Standard errors are clustered at the municipal level in parenthesis. * [**] {***} indicates statistical significance at a 10% [5%] {1%} confidence level. $N = 3636$ observations.

	Boredom	Loneliness	Irritability	Panic	Sleep	Stress	Worry	Frustration	Self-care
Rep. Number $R_0$	– 0.1935	0.3880***	– 0.4026***	– 0.1500	– 0.3720***	0.7096***	0.5702***	0.4822	0.3200***
	(0.1308)	(0.0670)	(0.1058)	(0.1220)	(0.0680)	(0.0663)	(0.0640)	(0.4292)	(0.0875)
2nd. Conf. Dummy	– 0.1391***	0.0081	– 0.1029**	– 0.0146	– 0.0096	0.0563*	0.1508***	0.6562***	0.0105
	(0.0470)	(0.0207)	(0.0485)	(0.0445)	(0.0233)	(0.0327)	(0.0253)	(0.1300)	(0.0219)

## Conclusions

We have estimated the effect of the implementations of lockdowns on the well-being and mental health of the Chilean population. We measure this effect by the changes in the volume of searches for keywords in Google Trends (GT) that are related to various topics: *Boredom*, *Loneliness*, *Irritability*, *Panic*, *Sleep*, *Stress*, *Worry*, *Frustration*, and *Self-care*. For this purpose, we exploit both the wide historical availability of GT data and especially the characteristics of the Chilean government’s response to the COVID-19 pandemic (the *Dynamic Quarantine*). In our sample, the government imposes weekly lockdowns on the population of some municipalities and lifts some from others that is determined by the authority’s evaluation of their epidemiological situation. Some municipalities were even subject to up to two distinct lockdowns during our sample period. The time and cross-sectional variation in the lockdowns provides us with the unique opportunity to estimate the population’s responses as differentiated by the socioeconomic status (SES) of the municipalities.

Perhaps unsurprisingly, lockdowns can have statistically significant and persistent effects on mental health. More interesting, we find strong evidence of SES induced heterogeneity in the population’s response to lockdown announcements: assuming that people carry out internet searches according to their mood, the population is more bored and more frustrated during lockdowns than during non-lockdown periods in which the levels of *Boredom* and *Frustration* are significantly lower for the population living in wealthy municipalities. On the other hand, the results also indicate that the population living in wealthy municipalities feel less lonely, less worried, and get less stressed during lockdowns. However, they do appear to experience higher levels of *Irritability* than the population from non-wealthy locations under lockdown. Finally, we find (weak) evidence that the population in wealthy municipalities is more concerned about *Self-care* during lockdowns, while there are no significant differences in the attention to *Panic* in GT searches by SES.

Our results indicate that SES heterogeneity should be accounted for in the design of the public policies aimed at providing support to individuals facing mental health difficulties triggered by the stringency of the measures taken to face the pandemic COVID-19. Neglecting such heterogeneity may lead to misleading conclusions about the size of the responses that are different for different SES, or the need to support certain segments of the population. Targeted public health responses must be implemented to address specific segments of the population with different mental health support needs. Although this issue may be of secondary importance for many countries or may seem difficult to implement, it should be kept in mind that municipalities are the smallest administrative units in the country, each with local health centers that can implement specific health policies.
